# MUC1 downregulation promotes TNF‐α‐induced necroptosis in human bronchial epithelial cells via regulation of the RIPK1/RIPK3 pathway

**DOI:** 10.1002/jcp.28148

**Published:** 2019-01-21

**Authors:** Huojun Zhang, Jiani Ji, Qian Liu, Shuyun Xu

**Affiliations:** ^1^ Department of Respiratory and Critical Care Medicine Key Laboratory of Pulmonary Diseases of Health Ministry, Tongji Hospital, Tongji Medical College, Huazhong University of Science and Technology Hubei China

**Keywords:** asthma, MUC1, necroptosis, RIPK1, RIPK3

## Abstract

MUC1 (mucin 1), a membrane‐tethered mucin glycoprotein, is highly expressed on the surface of respiratory epithelial cells and plays a key role in anti‐inflammatory and antiapoptotic responses against infections. However, little is known about the link between MUC1 and necroptosis in asthma. This study aimed to investigate the effects of MUC1 on TNF‐α‐induced necroptosis in human bronchial epithelial (16HBE) cells and the underlying molecular mechanism. Negative control and MUC1‐siRNA cells were treated with TNF‐α in the presence or absence of necrostatin‐1 (Nec‐1). Necroptosis was investigated using flow cytometry analyses, and the protein expression levels of MUC1, receptor‐interacting protein kinase‐1 (RIPK1), RIPK3, and phosphorylated RIPK1 were detected by western blot analysis. In addition, the interactions between RIPK and MUC1 were analyzed by coimmunoprecipitation. The results demonstrated that TNF‐α could induce necroptosis of 16HBE cells, and MUC1 expression was increased upon treatment with TNF‐α. The coimmunoprecipitation outcomes showed that MUC1 interacted with RIPK1 but not with RIPK3 in 16HBE cells, and the interaction was augmented by TNF‐α. Furthermore, MUC1 downregulation obviously increased the TNF‐α‐induced necroptosis of 16HBE cells and enhanced the expression of p‐RIPK1‐Ser166 and RIPK3, whereas these phenomena were partially attenuated by Nec‐1. These results may provide a new insight into the mechanism of severe asthma‐related necroptosis and lay a foundation for the future development of new anti‐inflammatory drugs for asthma.

## 1 INTRODUCTION

Asthma is a chronic inflammatory disease characterized by persistent airway inflammation, hyperresponsiveness, mucus secretion, and reversible airway obstruction (Saglani & Lloyd, 2015). Approximately 5–25% of asthmatics typically have a more severe disease, frequent and severe exacerbations and poor sensitivity to treatment even with high doses of steroids. These patients are said to have severe asthma and account for 50–80% of resources and health care costs spent on asthma (Hansbro et al., 2017). Recently, studies have shown that a variety of cells in the asthmatic airways, including eosinophils and epithelial cells, may undergo necrosis during the severe asthma (Naylor, 1962; Persson, & Uller, 2014; Uller, Persson, & Erjefalt, 2006). Kano, Almanan, Bochner, and Zimmermann (2013) demonstrated that Siglec‐8‐mediated necrosis in IL‐5‐activated eosinophils involves the loss of plasma membrane integrity and free extracellular granules (FEGs). FEGs produced cationic proteins which could cause epithelial shedding and then contribute to an exaggerated epithelial cell loss in severe asthma (Persson, 2014; Persson, & Uller, 2014). Increased levels of TNF‐α have been described in the airways of patients with severe asthma (Howarth et al., 2005). TNF‐α is a multipurpose cytokin: It can stimulate the generation of inflammatory mediators, induce cell death and, in contrast, promote cell proliferation in various cell types (Malaviya, Laskin, & Laskin, 2017). However, little is known regarding any association between TNF‐α and the occurrence of cell death in asthma.

Cell death in response to different insults can be loosely categorized as either apoptosis or a form of necrosis (Newton, 2015). Apoptosis was long considered as caspase‐dependent programmed cell death, while necrosis was thought to be an accidental, uncontrolled form of cell death. Over the past decade, a growing body of evidence has indicated that certain forms of necrosis are actually regulated by orchestrated pathways. These forms of necrosis have been termed necroptosis and have received much attention (Newton, 2015; Wegner, Saleh, & Degterev, 2016). Recent studies have suggested that TNF and related death ligands, can promote cells necroptosis. Within minutes after cells stimulation by TNF‐α, receptor‐interacting protein kinase‐1 (RIPK1) is recruited into the TNF‐R1 signaling complex and undergoes phosphorylation and ubiquitination. Phosphorylated RIPK1 is thought to have a crucial role in activating receptor‐interacting protein kinase‐3 (RIPK3) by binding through their shared RIP homotypic interaction motifs (RHIMs). Once activated, RIPK3 recruits its substrate, mixed lineage kinase domain‐like protein (MLKL), which appears to execute the process of necroptosis by targeting the plasma membrane (Cho et al., 2009; Geng et al., 2017; He et al., 2009; McQuade, Cho, & Chan, 2013; Moriwaki, Bertin, Gough, Orlowski, & Chan, 2015; Rodriguez et al., 2015; Zhang et al., 2009). In addition, scientists have discovered that necrostatin‐1 (Nec‐1), a highly specific small molecule inhibitor of RIPK1, efficiently inhibits the TNF‐α‐induced necroptosis by blocking necrosis‐specific RIPK1 phosphorylation at residues Ser14, Ser15, Ser161, and Ser166 (Ofengeim & Yuan, 2013).

The occurrence of necroptosis in asthma has only recently started to be explored (Cerps et al., 2018; Moreno‐Gonzalez, Vandenabeele, & Krysko, 2016). The levels of necroptosis markers, RIPK3 and pMLKL, were significantly increased in the lungs of virally induced animal model of asthma exacerbation, and IFN‐β knockout mice had higher levels of pMLKL compared to wild‐type mice (Cerps et al., 2018). However, the limitation of this study was that the cell types involved in asthma necroptosis were not identified.

Muc1 (murine)/MUC1 (human) is one of the membrane‐tethered mucins. It consists of two noncovalently associated polypeptide subunits, an N‐terminal extracellular subunit and a C‐terminal subunit containing its cytoplasmic tail (MUC1‐CT). MUC1‐CT can shed from the cell membrane and together with some of its binding partners, kinases and adapter proteins, induce signal transduction events (Apostolopoulos, Stojanovska, & Gargosky, 2015). Previous studies unveiled that MUC1 served an anti‐inflammatory role in the airways in response to a variety of infectious insults (Kato, Hanss, et al., 2017; Kato, Lillehoj, Lu, & Kim, 2017; Lu et al., 2006). In addition, Ahmad et al. (2007) showed that MUC1 was recruited to the TNF‐R1 complex upon stimulation by TNF‐α and directly bound to the IκB kinase complex, resulting in reduced cellular apoptosis. MUC1 was also found to prevent activation of the death receptor‐induced apoptosis by binding to the Fas‐associated death domain (FADD; Agata et al., 2008). Although MUC1 has been suggested to have a significant role in apoptosis, little is known about the link between MUC1 and necroptosis in asthma.

The aim of the present study was to determine whether silencing the MUC1 expression increases TNF‐α‐induced necroptosis in the human bronchial epithelial (16HBE) cells in vitro. Furthermore, we aimed to elucidate the underlying molecular mechanisms, specifically, whether this effect was completed through the RIPK1/RIPK3 pathway.

## 2 MATERIALS AND METHODS

### 2.1 Cell culture

The 16HBE cell line was purchased from Wuhan Boster Biotech Co. Ltd. (People’s Republic of China), and cultured in RPMI 1640 medium (Gibco Laboratories, Grand Island, NY) supplemented with 10% fetal bovine serum and 1% penicillin and streptomycin in a 5% CO_2_ atmosphere at 37℃. The study was approved by the Ethics Committee of Tongji Hospital, Tongji Medical College, Huazhong University of Science and Technology.

### 2.2 Cell death assays

TNF‐α (Peprotech, Rocky Hill, CT)‐induced cellular death in 16HBE cells was investigated using the Annexin V/PI Apoptosis Detection Kit (Key Gen, Nanjing, China). Following incubation of cells with TNF‐α (300 ng/ml), they were washed in phosphate buffered saline and resuspended in 500 μl of binding buffer containing 5 μl of annexin V‐FITC and 5 μl of PI. Afterwards, cells were incubated for 15 min at room temperature in the dark. Samples were analyzed by flow cytometry using a Becton Dickinson LSR flow cytometer. The data were analyzed with Cell Quest software. Annexin V(+)/PI(+) cells in upper right quadrant were considered as necrotic.

### 2.3 MUC1‐small interfering RNA (siRNA) transfection

MUC1 siRNA and nontargeting negative control siRNA were synthesized by Viewsolid Biotech (Beijing, China). The target sequences were as follows: MUC1‐siRNA sense 5′‐GUU CAG UGC CCA GCU CUA CTT‐3′, and MUC1‐siRNA antisense 5′‐GUA GAG CUG GGC ACU GAA CTT‐3′. SiRNAs were transfected into 16HBE cells using Lipofectamine 2000 according to the manufacturer’s protocol (Invitrogen, Carlsbad, CA). Following incubation for 48 hr, MUC1 protein levels were determined by western blot analysis.

### 2.4 Immunoblot and coimmunoprecipitation analyses

Extracts of 16HBE cells were prepared with the lysis buffer, and the protein concentration in homogenates was measured by the BCA Protein Assay Kit (Aspen Biological, Wuhan, China). Western blots were performed following standard procedures. Briefly, equal protein aliquots were subjected to 10% sodium dodecyl sulfate polyacrylamide gel electrophoresis (SDS‐PAGE) gel, and then the proteins were transferred to polyvinylidene fluoride membranes, which were incubated with primary antibodies against RIPK1 (Abnova, Taiwan, China; MAB0836), p‐RIPK1 (Cell Signaling Technology, Danvers, MA; 65746), RIPK3 (Proteintech, Chicago, IL; 17563‐1‐AP), and MUC1‐CT (Thermo Fisher Scientific, Waltham, MA; MA5‐11202). The intensity of individual bands was quantified using ImageJ (NIH Image, Bethesda, MD). For coimmunoprecipitation assays, equal protein aliquots of cell lysates were incubated overnight at 4°C with immunoprecipitating antibodies against RIPK1 (Abnova; MAB0836), RIPK3 (Proteintech; 17563‐1‐AP), MUC1‐CT (Novus, Colorado; NBP1–60046), or with isotype‐matched nonimmune IgG. Protein A/G agarose beads (Abmart, Shanghai, China; A10001) were added. The beads were washed five times at 4°C with lysis buffer and eluted by boiling in SDS‐PAGE loading buffer. Immunoprecipitated proteins were separated by SDS‐PAGE and subjected to immunoblot analysis for the protein of interest.

### 2.5 Statistical analysis

Statistical analysis was performed using GraphPad Prism V6.0 (GraphPad Software Inc., San Diego, CA), and the data are presented as the mean ± *SD*. A two‐tailed Student *t*‐test was used for comparison between two groups and one‐way analysis of variance for more than two groups. A value of *p* < 0.05 was considered as an indicator of a statistically significant difference.

## 3 RESULTS

### 3.1 MUC1 downregulation promotes TNF‐α‐induced necroptosis in 16HBE cells

**Figure 1:**
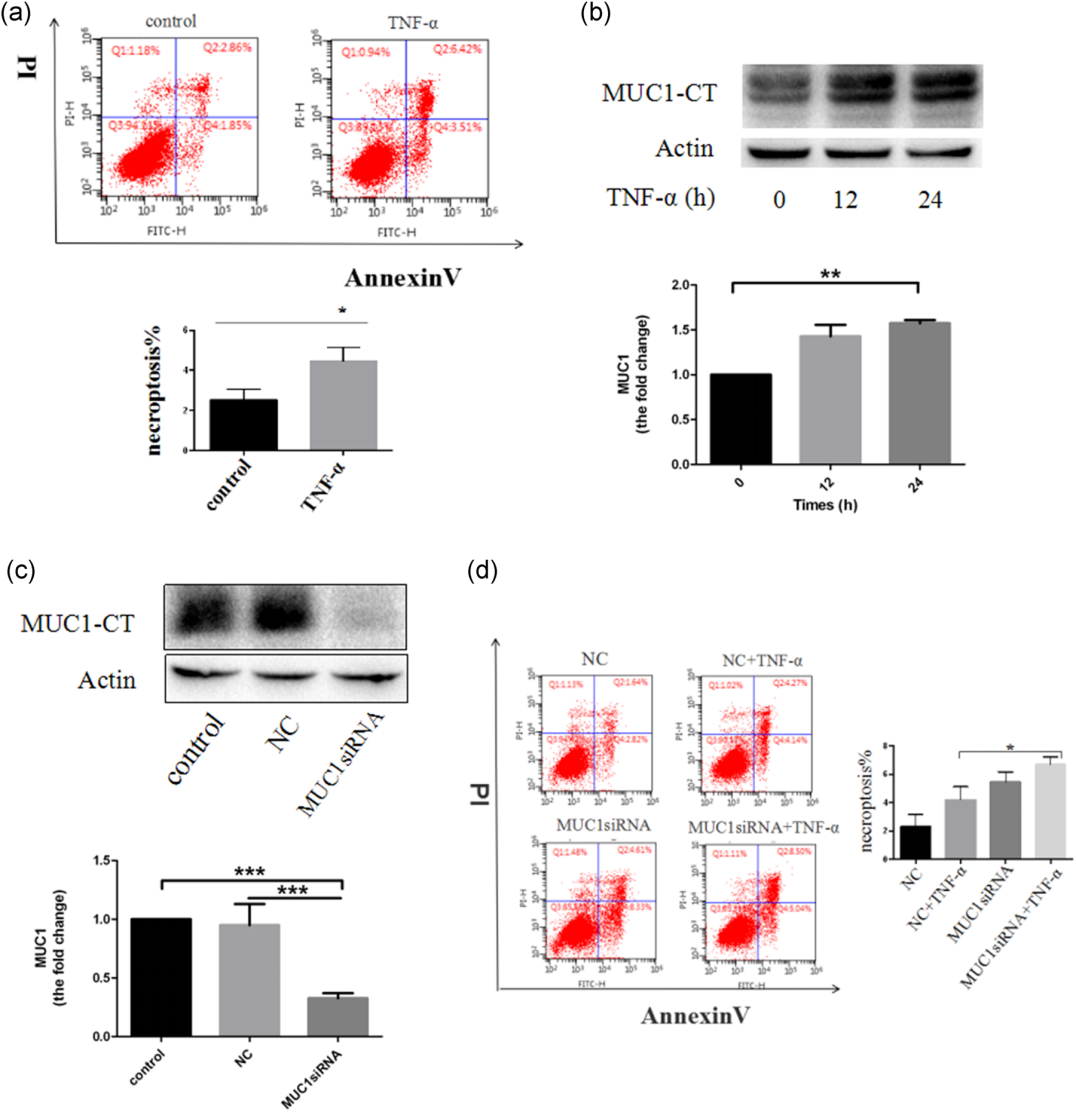
MUC1 downregulation promotes TNF‐α‐induced necroptosis in 16HBE cells. (a) Flow cytometry analysis for necroptosis detection of 16HBE cells treated with TNF‐α (300 ng/ml) for 24 hr. (b) Western blot detected MUC1 protein expression with the TNF‐α (300 ng/ml) treatment for the indicated times. (c) After silencing MUC1 expression by siRNA transfection for 48 hr in 16HBE cells, protein expression levels of MUC1 were determined by western blot analysis. (d) Flow cytometry was conducted to detect the cell necroptosis after 16HBE cells transfected with MUC1‐siRNA or NC‐siRNA were treated for 24 hr with TNF‐α. Data are expressed as means ± *SD* (*n* = 3) and were analyzed by Student *t*‐test or one‐way analysis of variance, * *p* < 0.05; ** *p* < 0.01; *** *p* < 0.001. NC group (transfected with NC siRNA); MUC1‐siRNA group (transfected with MUC1‐siRNA). 16HBE: human bronchial epithelial; NC: negative control; siRNA: small interfering RNA; TNF‐α: tumor necrosis factor‐α [Color figure can be viewed at wileyonlinelibrary.com]

First, we determined whether TNF‐α could initiate necroptosis in 16HBE cells after treatment with TNF‐α (300 ng/ml). Our findings indicated that TNF‐α induced 16HBE cell necroptosis compared with control group (Figure 1a). Next, we wondered whether MUC1 is involved in TNF‐α‐induced necroptosis in 16HBE cells. We used MUC1 antibody to detect MUC1 protein expression. Western blot analysis showed that MUC1 expression was increased after TNF‐α stimulation for the indicated time periods (Figure 1b). Therefore, we further decided to investigate whether MUC1 downregulation affects necroptosis by transfecting 16HBE cells with MUC1‐siRNA or negative control siRNA. As shown in Figure 1c, cells transfected with MUC1‐siRNA displayed a significant reduction in the expression levels of MUC1 protein compared with the negative control siRNA (Figure 1c). Furthermore, MUC1 knockdown effectively increased necroptosis following TNF‐α stimulation compared with that of cells transfected with the negative control siRNA (Figure 1d). Collectively, these data suggest that MUC1 deficiency contributes to TNF‐α‐induced necroptosis by 16HBE cells.

### 3.2 MUC1 binds RIPK1 to mediate the RIPK1/RIPK3 pathway

**Figure 2:**
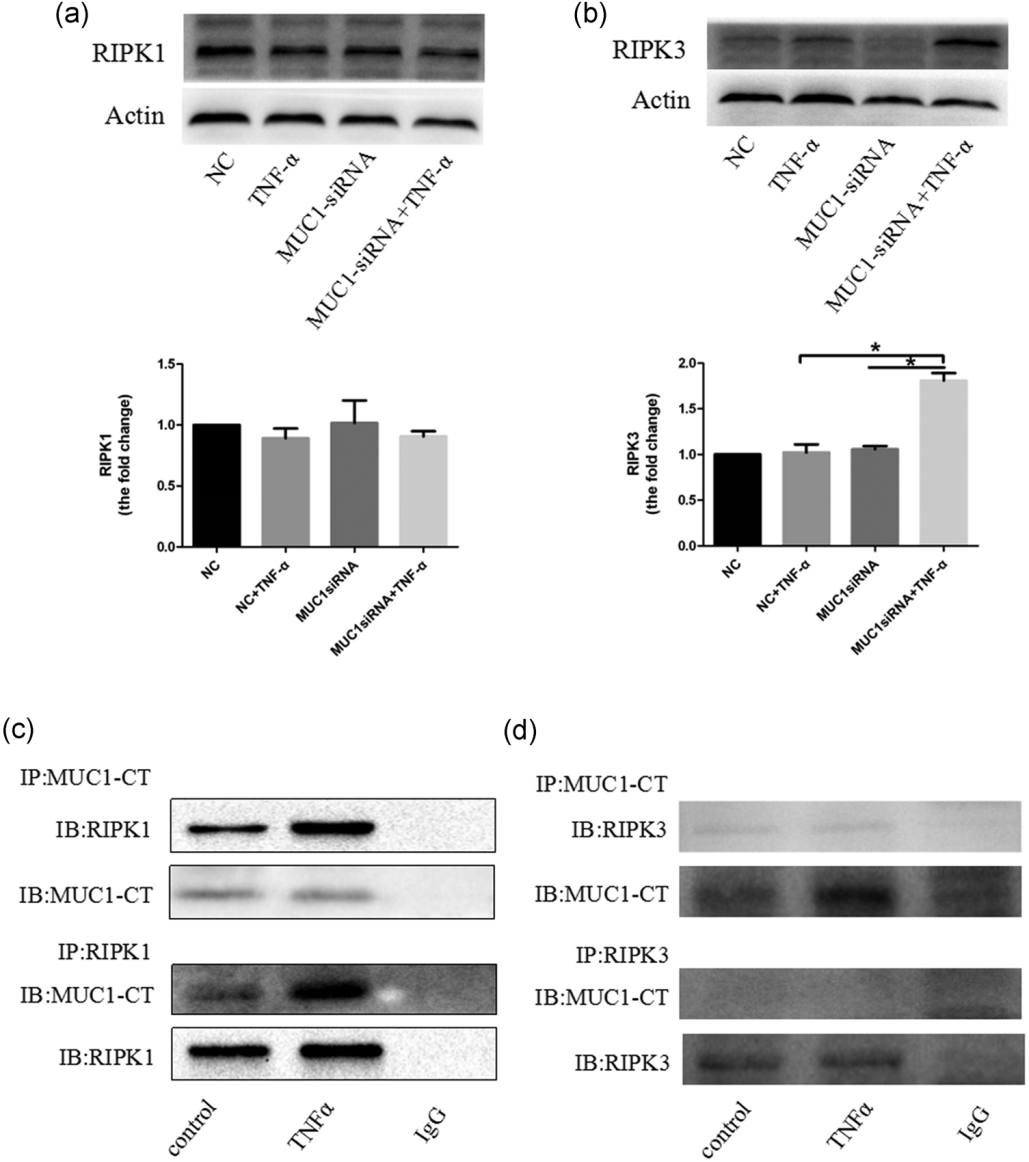
MUC1 binds RIPK1 to mediate the RIPK1/RIPK3 pathway. (a,b) MUC1 knockdown by MUC1‐siRNA or NC‐siRNA were assayed in 16HBE cells following stimulation with TNF‐α (300 ng/ml). After 24 hr of stimulation, RIPK3 and RIPK1 protein levels were measured by western blot analysis. (c,d) Normal 16HBE cells were treated with TNF‐α for 24 hr, and the interaction between MUC1‐CT and RIPK1/RIPK3 were confirmed by immunoprecipitation. Data are expressed as means ± *SD* and were analyzed by one‐way analysis of variance, * *p* < 0.05. NC group (transfected with NC siRNA); MUC1‐siRNA group (transfected with MUC1‐siRNA). 16HBE: human bronchial epithelial; IB: immunoblotting; IP: immunoprecipitation; RIPK1: receptor‐interacting protein kinase‐1; RIPK3: receptor‐interacting protein kinase‐3; NC: negative control; siRNA: small interfering RNA; TNF‐α: tumor necrosis factor‐α

The RIPK1 and RIPK3 signaling pathways have been strongly involved in necroptosis. To understand the effect of MUC1 on the activation of these two pathways, the expression levels of RIPK1 and RIPK3 were examined. Western blot analysis revealed that MUC1‐siRNA cells showed a significant increase in RIPK3 following TNF‐α stimulation compared with negative control group (Figure 2b). However, it could not upregulate the expression of RIPK1 (Figure 2a). Previous studies revealed that MUC1‐CT could bind to multiple kinases, thus reducing cellular apoptosis in TNF‐α signaling. To understand whether MUC1‐CT can form a stable complex with RIPK1 or RIPK3, we used coimmunoprecipitation to detect MUC1 and RIPK1/RIPK3 interaction. The results uncovered that MUC1 interacted with RIPK1 in 16HBE cells, which was augmented by TNF‐α, but not with RIPK3 (Figure 2c,d). Altogether, these results indicate that MUC1 downregulation promotes TNF‐α‐induced necroptosis in 16HBE cells by mediating the RIPK1/RIPK3 pathway.

### 3.3 Silencing of MUC1 expression potentiates TNF‐α‐induced phosphorylation of RIPK1 at Ser166

**Figure 3:**
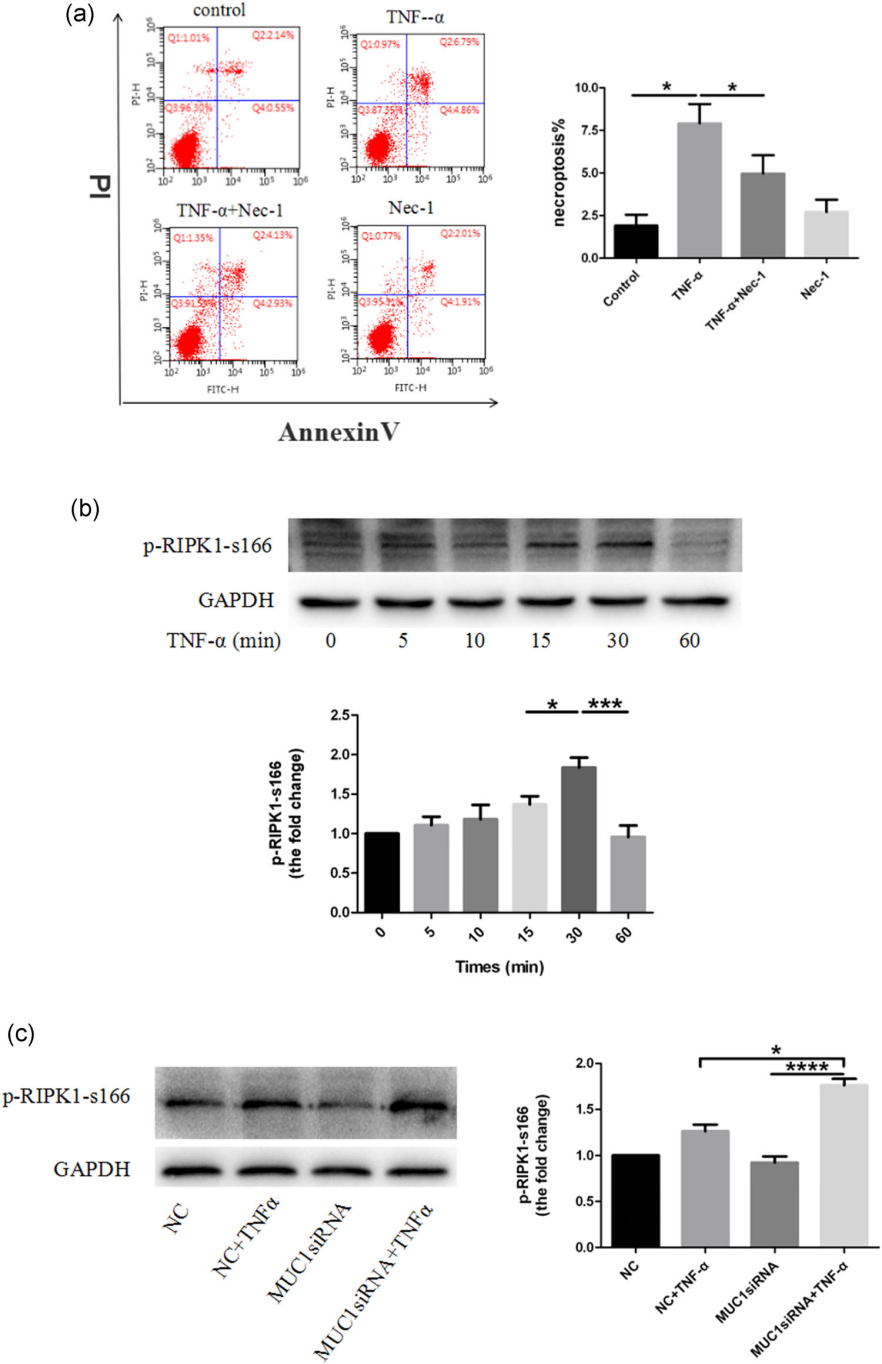
Silencing of MUC1 expression potentiates TNF‐α‐induced phosphorylation of RIPK1 at Ser166. (a) 16HBE cells were or were not treated with Nec‐1 (50 μM) for 2 hr followed by an addition of TNF‐α (300 ng/ml). After 24 hr, cell death was analyzed by annexin V/PI double staining. (b) 16HBE cells were treated with TNF‐α (300 ng/ml) for the indicated times and p‐RIPK1‐s166 was analyzed by western blot. (c) MUC1 knockdown by MUC1‐siRNA or NC‐siRNA were assayed in 16HBE cells following stimulation with TNF‐α (300 ng/ml). After 30 min of stimulation, p‐RIPK1‐s166 protein levels were measured by western blot analysis. Data are expressed as means ± *SD* (*n* = 3) and were analyzed by one‐way analysis of variance, * *p* < 0.05; *** *p* < 0.001; **** *p* < 0.0001. NC group (transfected with NC‐siRNA); MUC1‐siRNA group (transfected with MUC1‐siRNA). 16HBE: human bronchial epithelial; NC: negative control; Nec‐1: necrostatin‐1; RIPK1: receptor‐interacting protein kinase‐1; siRNA: small interfering RNA; TNF‐α: tumor necrosis factor‐α [Color figure can be viewed at wileyonlinelibrary.com]

The previous results demonstrated that MUC1 downregulation increased the expression of RIPK3 but not RIPK1 and that MUC1 could bind RIPK1. Therefore, we focused on whether MUC1 downregulation alters the phosphorylation of RIPK1 expression. First, we used Nec‐1 to treat 16HBE cells, and the results proved that Nec‐1 efficiently reduced TNF‐α‐induced necroptosis in 16HBE cells (Figure 3a), which indicated that phosphorylation of RIPK1 participated in TNF‐α‐induced necroptosis. Second, we decided to detect the phosphorylation of RIPK1 at Ser166 (p‐RIPK1‐s166) because it is easily achieved. Our data suggested that phosphorylation of RIPK1 at Ser166 by TNF‐α was an early and transient event, as it was the most‐pronounced within 30 min and was lost after 60 min (Figure 3b). Thus, we chose the 30‐min stimulation with TNF‐α for further experiments. Next, western blot analysis showed that knockdown of MUC1 by siRNA led to upregulation of p‐RIPK1‐s166 compared with the NC group (Figure 3c). These findings corroborate the idea that MUC1 downregulation at least in part, accelerates necroptosis by modulating the phosphorylation of RIPK1 at Ser166.

### 3.4 NEC‐1 partially blocks MUC1‐siRNA regulated necroptosis induced by TNF‐α stimulation

**Figure 4:**
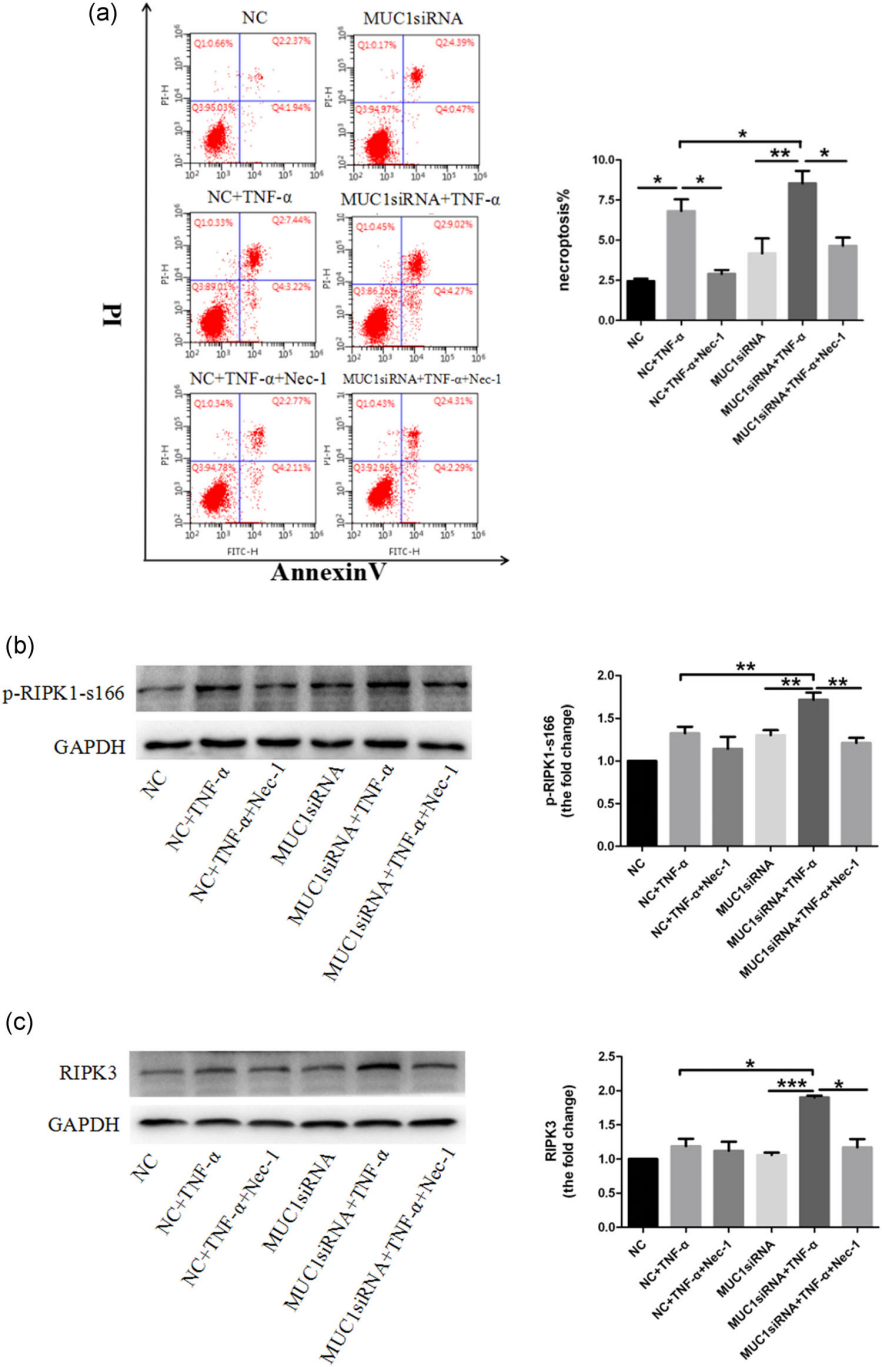
Nec‐1 partially blocks MUC1‐siRNA‐regulated necroptosis induced by the TNF‐α. stimulation. (a) MUC1 knockdown MUC1‐siRNA or NC‐siRNA was assayed in 16HBE cells following stimulation with TNF‐α (300 ng/ml) in the presence or absence of 50 μM Nec‐1 (added 2 hr before stimulus). After 24 hr of stimulation, cell necroptosis was measured by annexin V/PI double staining. (b) The MUC1‐siRNA cells and NC cells were or were not treated with Nec‐1 (50 μM) for 2 hr followed by an addition of TNF‐α (300 ng/ml). After 30 min, p‐RIPK1‐s166 expression levels were determined by immunoblotting. (c) The MUC1‐siRNA cells and NC cells were treated with Nec‐1 (50 μM) for 2 hr, following stimulation with TNF‐α (300 ng/ml) for 24 hr, and RIPK3 expression was detected by western blot analysis. Data are expressed as means ± *SD* (*n* = 3) and were analyzed by one‐way analysis of variance, * *p* < 0.05; ** *p* < 0.01; ***p < 0.001. NC group (transfected with negative control siRNA); MUC1‐siRNA group (transfected with MUC1‐siRNA). 16HBE: human bronchial epithelial; RIPK1: receptor‐interacting protein kinase‐1; RIPK3: receptor‐interacting protein kinase‐3; NC: negative control; Nec‐1: necrostatin‐1; siRNA: small interfering RNA; TNF‐α: tumor necrosis factor‐α [Color figure can be viewed at wileyonlinelibrary.com]

**Figure 5:**
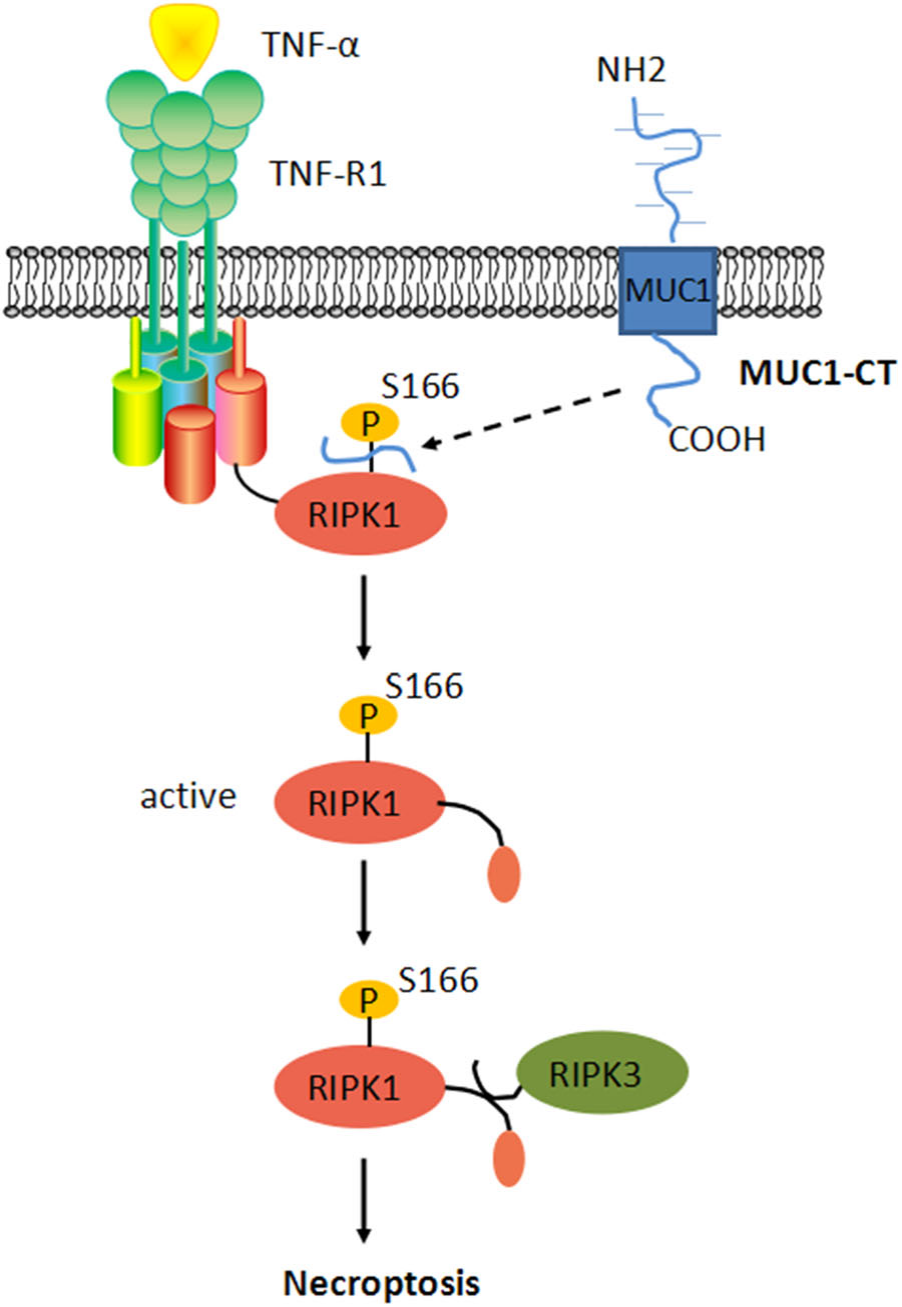
MUC1 modulates TNF‐α‐induced 16HBE cells necroptosis. MUC1‐CT binds RIPK1 when it is released from an epithelial cell in response to TNF‐α. Then, it affects the phosphorylation of RIPK1 at Ser166. Upon activation, RIPK1 recruits RIPK3 to necrosome to initiate necroptosis. 16HBE: human bronchial epithelial; RIPK1: receptor‐interacting protein kinase‐1; RIPK3: receptor‐interacting protein kinase‐3; TNF‐α: tumor necrosis factor‐α [Color figure can be viewed at wileyonlinelibrary.com]

To further verify whether Nec‐1 inhibits 16HBE cells necroptosis caused by MUC1 downregulation in this study, we used the specific RIPK1 inhibitor Nec‐1 to treat 16HBE cells. The results indicated that Nec‐1 significantly protected TNF‐α‐induced necroptosis in 16HBE cells regardless of the MUC1 presence. However, the MUC1‐siRNA + TNF‐α + Nec‐1 group still showed higher cell necroptosis in comparison with the NC + TNF‐α + Nec‐1 group (Figure 4a), which indicated that Nec‐1 partially blocked MUC1‐siRNA increased necroptosis induced by TNF‐α stimulation. In addition, we also found that Nec‐1 could decrease the p‐RIPK1‐s166 and RIPK3 expression in MUC1‐siRNA cells treated with TNF‐α compared with negative control cells (Figures 4b,c and 5). Taken together, these data suggest that MUC1 downregulation promotes TNF‐α‐induced necroptosis in 16HBE cells, accompanied by an increase in protein levels of p‐RIPK1‐s166 and RIPK3, which could be partially blocked by Nec‐1.

## 4 DISCUSSION

In this study, we provided the first evidence that TNF‐α could induce 16HBE cells necroptosis accompanied by the upregulation of MUC1. Furthermore, we demonstrated that MUC1 downregulation contributed to cell necroptosis through a mechanism that involved the RIPK1/RIPK3 signaling and it could be partially blocked by Nec‐1.

Asthma is characterized by airway inflammation, remodeling and hyperresponsiveness (Saglani & Lloyd, 2015). Bronchial epithelial cells (BECs) are the first line of defense against pathogens and inhaled allergens that may cause asthma (Lambrecht & Hammad, 2012). To date, some studies indicated that the loss of BEC integrity, which may be explained by cell death, is a hallmark of asthma pathogenesis (Chanez, 2005; Holgate, 2011; Martínez‐Girón & van Woerden, 2010). Recently, certain forms of cell death are accepted as being actually regulated by orchestrated pathways, such as necroptosis (Newton, 2015; Wegner et al., 2016). Although an occurrence of necroptosis in asthma has been identified, the exact cell types involved have not been clearly identified. In the present study, we have indeed found that TNF‐α could induce necroptosis in 16HBE cells. In addition, studies demonstrated that MUC1 could regulate the IκB kinase complex and Fas‐associated death domain to inhibit cell apoptosis in response to TNF‐α (Agata et al., 2008; Ahmad et al., 2007). Similarly, we observed that MUC1 downregulation obviously increased the TNF‐α‐induced 16HBE cell necroptosis. These results suggest that MUC1 can serve a possible protective role both in antiapoptotic and antinecroptotic.

To elucidate the mechanisms responsible for the promotion of TNF‐α‐induced 16HBE cell necroptosis by MUC1 downregulation, we examined the RIPK1/RIPK3 signaling because its role is widely accepted in the regulation of necroptosis (Newton, 2015; Wegner et al., 2016). RIPK3 has emerged as the common pro‐necrotic protein kinase, is crucial in determining the cell death model. Recent evidence indicate that cells deficient in RIPK3 were protected from necroptosis (He et al., 2009). In this study, we found that MUC1 deficiency significantly improved RIPK3 but not RIPK1 expression in 16HBE cells induced by TNF‐α. Furthermore, some studies have shown that MUC1 could bind IκB kinase and FADD to regulate TNF‐α‐induced apoptosis (Agata et al., 2008; Ahmad et al., 2007). We thus hypothesized that MUC1 might bind RIPK1 or RIPK3. Our results demonstrate that MUC1 exhibited a strong interaction with RIPK1 in 16HBE cells, augmented by TNF‐α, yet only a weak interaction with RIPK3 when immunoprecipitation with anti‐MUC1‐CT Ab coprecipitated RIPK3. A possible reason is that RIPK1, but not RIPK3, bears a death domain (DD) that can interact with the FADD to form a complex on the cytoplasmic domain, and could recruit RIPK3 via their shared RHIMs (Agata et al., 2008). These results suggested that MUC1 could bind RIPK1 to mediate the RIPK1/RIPK3 pathway. However, further studies are needed to understand whether MUC1 directly binds RIPK1 in 16HBE cells.

In recent years, many studies have confirmed that TNF‐α induces cell necroptosis partly via TNF‐α‐mediated phosphorylation and ubiquitination of RIPK1. Phosphorylated RIPK1 is thought to have a crucial role in activating RIPK3 (Cho et al., 2009; Geng et al., 2017; He et al., 2009; McQuade et al., 2013; Moriwaki et al., 2015; Rodriguez et al., 2015; Zhang et al., 2009). Furthermore, studies have also discovered that Nec‐1 efficiently inhibits the TNF‐α‐induced necroptosis by blocking necrosis‐specific RIPK1 phosphorylation at residues Ser14, Ser15, Ser161, and Ser166 (Ofengeim & Yuan, 2013). We thus speculated that MUC1 might alter the phosphorylation of RIPK1 in TNF‐α‐induced necroptosis. In this study, our data revealed that Nec‐1 inhibited the TNF‐α‐induced necroptosis, indicating that the phosphorylation of RIPK1 was involved in TNF‐α‐induced necroptosis. More interestingly, knockdown of MUC1 significantly increased the expression of p‐RIPK1‐s166. To further support of this finding, we wondered that whether Nec‐1 could antagonize the MUC1 downregulation‐related aggravation of TNF‐α‐induced necroptosis. In this study, Nec‐1 could significantly inhibit TNF‐α‐induced necroptosis in 16HBE cells with MUC1 downregulation. However, the MUC1‐siRNA + TNF‐α + Nec‐1 group still showed higher cell necroptosis in comparison with the NC + TNF‐α + Nec‐1 group, suggesting that MUC1 participation in TNF‐α‐induced 16HBE cells necroptosis might not only include phosphorylation but also ubiquitination. In addition, we also found that Nec‐1 could decrease the p‐RIPK1‐s166 and RIPK3 expression in MUC1‐siRNA cells treated with TNF‐α. Consistently, these outcomes are similar with previous findings that Nec‐1 impeded TNF‐α‐induced necroptosis by blocking necrosis‐specific RIPK1 phosphorylation at Ser166 (Jaco et al., 2017; Menon et al., 2017). Collectively, these data suggest that Nec‐1 can antagonize TNF‐α‐induced necroptosis in 16HBE cells with MUC1 downregulation and thus, furtherly support that MUC1 as a novel protective molecule in cell necroptosis on asthma through mediating the p‐RIPK1‐s166 expression.

## 5 CONCLUSION

In conclusion, we have demonstrated that MUC1 downregulation aggravates TNF‐α‐induced necroptosis in 16HBE cells by mediating the RIPK1/RIPK3 pathway, and the effect could be partially blocked by Nec‐1. This may provide a new insight into the effects of severe asthma on necroptosis and lay a foundation for the future development of new anti‐inflammatory drugs for asthma.

## CONFLICTS OF INTEREST

The authors declare that there are no conflicts of interest.
